# Supplementary material for: Causal Relationship between Aging and Anorexia Nervosa: A White-Matter-Microstructure-Mediated Mendelian Randomization Analysis
Source: Biomedicines. 2024 Aug 16;12(8):1874. doi: 10.3390/biomedicines12081874 (PMC11351342; doi:10.3390/biomedicines12081874)
Supplement: Supplementary file 1 [file biomedicines-12-01874-s001.zip › Supplementary Figure/Figure S4/Singe SNP plot of MR analysis for TL to ubm-b-1956.pdf]

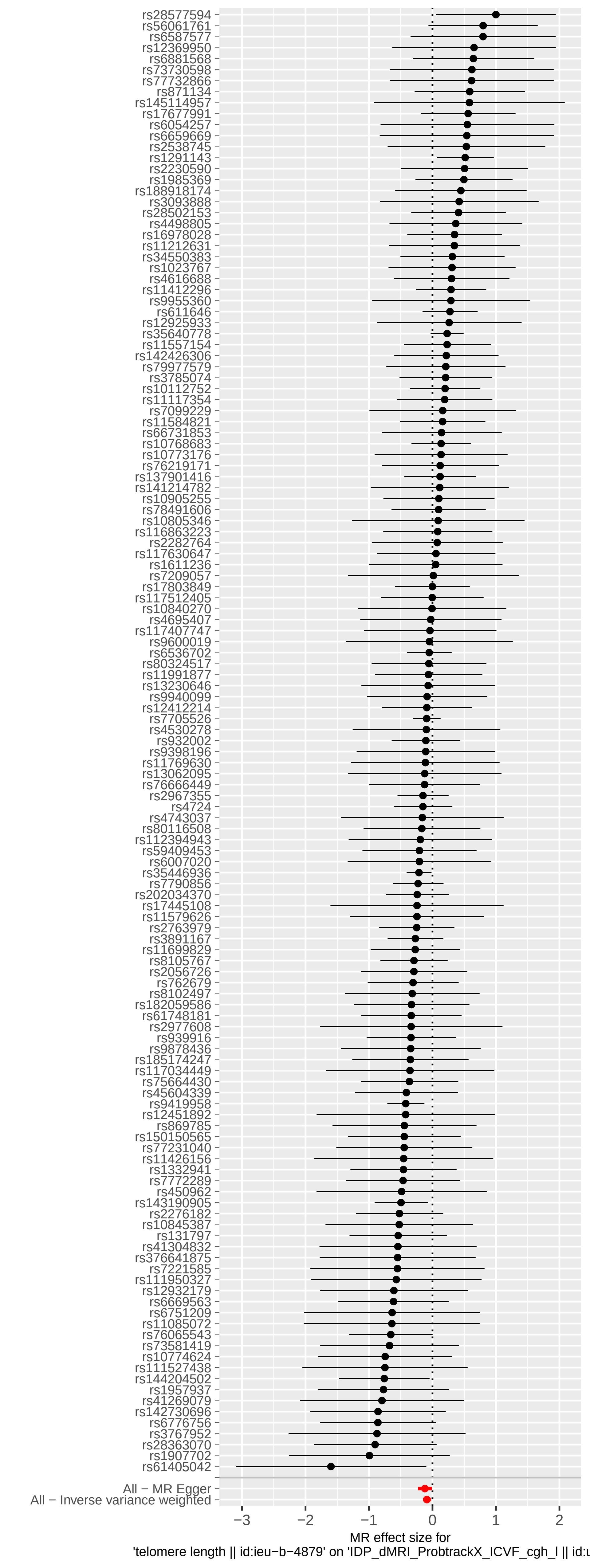
rs28577594
rs56061761
rs6587577
rs12369950
rs6881568
rs73730598
rs77732866
rs871134
rs145114957
rs17677991
rs6054257
rs6659669
rs2538745
rs1291143
rs2230590
rs1985369
rs188918174
rs3093888
rs28502153
rs4498805
rs16978028
rs11212631
rs34550383
rs1023767
rs4616688
rs11412296
rs9955360
rs611646
rs12925933
rs35640778
rs11557154
rs142426306
rs79977579
rs3785074
rs10112752
rs11117354
rs7099229
rs11584821
rs66731853
rs10768683
rs10773176
rs76219171
rs137901416
rs141214782
rs10905255
rs78491606
rs10805346
rs116863223
rs2282764
rs117630647
rs1611236
rs7209057
rs17803849
rs117512405
rs10840270
rs4695407
rs117407747
rs9600019
rs6536702
rs80324517
rs11991877
rs13230646
rs9940099
rs12412214
rs7705526
rs4530278
rs932002
rs9398196
rs11769630
rs13062095
rs76666449
rs2967355
rs4724
rs4743037
rs80116508
rs112394943
rs59409453
rs6007020
rs35446936
rs7790856
rs202034370
rs17445108
rs11579626
rs2763979
rs3891167
rs11699829
rs8105767
rs2056726
rs762679
rs8102497
rs182059586
rs61748181
rs2977608
rs939916
rs9878436
rs185174247
rs117034449
rs75664430
rs45604339
rs9419958
rs12451892
rs869785
rs150150565
rs77231040
rs11426156
rs1332941
rs7772289
rs450962
rs143190905
rs2276182
rs10845387
rs131797
rs41304832
rs376641875
rs7221585
rs111950327
rs12932179
rs6669563
rs6751209
rs11085072
rs76065543
rs73581419
rs10774624
rs111527438
rs144204502
rs1957937
rs41269079
rs142730696
rs6776756
rs3767952
rs28363070
rs1907702
rs61405042
All − MR Egger
All − Inverse variance weighted
−3
−2
−1
0
1
2
MR effect size for
'telomere length || id:ieu−b−4879' on 'IDP_dMRI_ProbtrackX_ICVF_cgh_l || id:u
